# Stimuli-Responsive Double Single-Atom Catalysts for Parallel Catalytic Therapy

**DOI:** 10.3390/pharmaceutics15041217

**Published:** 2023-04-11

**Authors:** Tushuai Li, Yue Gu, Lisha Yu, Shenglong Zhu, Jie Zhang, Yongquan Chen

**Affiliations:** 1 School of Food Science and Technology, Jiangnan University, Wuxi 214013, China; 2 Wuxi School of Medicine, Jiangnan University, Wuxi 214013, China; 3 Wuxi Translational Medicine Research Center and Jiangsu Translational Medicine Research Institute Wuxi Branch, Wuxi 214013, China; 4 Key Laboratory of Anti-Inflammatory and Immune Medicine, Ministry of Education, Institute of Clinical Pharmacology, Anhui Medical University, Hefei 230032, China; 5 School of Biology and Food Engineering, Changshu Institute of Technology, Suzhou 215500, China

**Keywords:** tumor microenvironment, single-atom catalysts, reactive oxygen species, synergistic antitumor

## Abstract

Tumor microenvironment (TME)-induced nanocatalytic therapy is a trending strategy for tumor-targeting therapy, but the low catalytic efficiency remains to limit its therapeutic effect. The single-atom catalysts (SACs) appear as a novel type of nanozymes that possesses incredible catalytic activity. Here, we developed PEGylated manganese/iron-based SACs (Mn/Fe PSACs) by coordinating single-atom Mn/Fe to nitrogen atoms in hollow zeolitic imidazolate frameworks (ZIFs). Mn/Fe PSACs catalyze cellular hydrogen peroxide (H_2_O_2_) converting to hydroxyl radical (•OH) through a Fenton-like reaction; it also enhances the decomposition of H_2_O_2_ to O_2_ that continuously converts to cytotoxic superoxide ion (•O_2_^−^) via oxidase-like activity. Mn/Fe PSACs can reduce the depletion of reactive oxygen species (ROS) by consuming glutathione (GSH). Here, we demonstrated the Mn/Fe PSACs-mediated synergistic antitumor efficacy among in vitro and in vivo experiments. This study proposes new promising single-atom nanozymes with highly efficient biocatalytic sites and synergistic therapeutic effects, which will give birth to abundant inspirations in ROS-related biological applications in broad biomedical fields.

## 1. Introduction

Cancer has become a deadly threat to global human health nowadays, and the complexity and diversity of tumors still require more advanced and effective treatments. For decades, nanozymes with peroxidase (POD)-like activity have demonstrated their great potential for converting hydrogen peroxide (H_2_O_2_) and oxygen (O_2_) into toxic reactive oxygen species (ROS) [1,2,3], for instance, singlet oxygen (^1^O_2_) [4], superoxide ion (•O_2_^−^) [5], or hydroxyl radical (•OH) [6]. ROS is known as a super oxidant, which has an anti-tumor effect on the immune system, and ROS can kill cancer cells by destroying DNA, proteins, cell membranes, etc., [7,8,9,10] The excessive products of H_2_O_2_ decomposition in the tumor microenvironment (TME) provide raw materials for enhancing the effect of chemodynamic therapy (CDT) on tumors by mediating Fenton-like reactions [11,12]. A more advantageous point is that the H_2_O_2_ deficiency in normal cells does not cause severe Fenton reaction, which means minor damage to normal tissues [13,14,15]. Some researchers pointed out that tumor cells are more sensitive to ROS than normal cells, which may be related to their different antioxidant mechanisms [16,17]. Therefore, utilizing highly generated ROS to eliminate cancer cells would be an ideal antitumor strategy. However, it does need to be noted that the process of anaerobic glycolysis produces an abnormal excess of glutathione (GSH) in the TME, which can greatly reduce the catalytic activity of nanozyme and affect the therapeutic effect [18,19,20,21]. Therefore, reducing GSH levels in the TME is crucial to enhance the effectiveness of CDT against tumors.

Single-atom catalysts (SACs) allow for the maximal presence of accessible active sites and may confer unique catalytic properties unattainable by their non-atomically dispersed counterparts [22,23,24,25]. The well-defined atomic-level dispersion provides maximum atom utilization efficiency, favoring atom economy for metal usage [26,27,28,29]. Their unique structure and coordination environment enhances catalytic activity in reactions with superior stability of materials [30,31,32,33]. Therefore, the construction of SACs will be an effective strategy for organismal biochemical reactions. Recently, economical SACs composed of Fe-N_4_ moieties have been listed as one of the most promising candidates for excellent activity because of their lowest cost and ultrahigh metal utilization [34,35,36,37]. However, the structure of Fe-N_4_ exists in a state of symmetrical electron distribution, resulting in ideal adsorption energy for O_2_ intermediates. Herein, establishing an adjacent metal center to regulate the electron localization of Fe-N_4_ would be a promising approach to promote ROS generation.

**Scheme 1:**
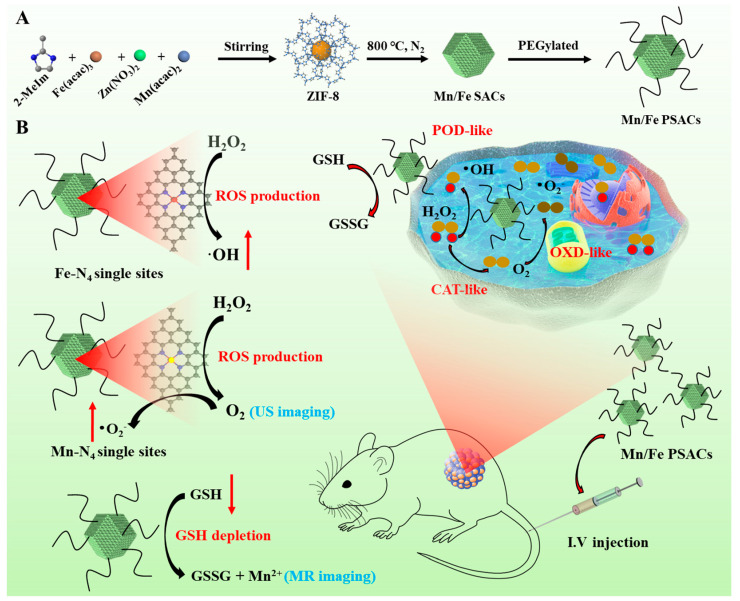
Schematic Illustration for (**A**) the synthesis process of Mn/Fe SACs and (**B**) antitumor treatment of Mn/Fe SACs.

In this study, PEGylated nanocatalysts of SACs (Mn/Fe PSACs) with the co-presence of Fe-N_4_ and Mn-N_4_ moieties were synthesized, in which SACs have an efficient ability to catalyze POD-like reactions, producing abundant ROS kills cancer cells (Scheme 1). The single Mn/Fe atoms coordinated to nitrogen show high Fenton activity in hollow zeolite imidazole frameworks (ZIF-8s). With this advantage, Mn/Fe PSACs convert cellular H_2_O_2_ into •OH through a Fenton-like reaction, while the decomposed product–O_2_ can be continuously catalyzed into cytotoxic •O_2_^−^ through oxidase-like activity. Moreover, Mn/Fe PSACs also exhibited GSH-depleting properties, which could enhance the therapeutic effect of ROS-eliminating cancer. The excellent chemodynamic performance and magnetic resonance contrast imaging capabilities of Mn/Fe PSACs also support superior tumor suppression. Above all, Mn/Fe PSACs can serve as super diagnostic and therapeutic nanoagents, which have great potential for biomedical and clinical treatments in the future.

## 2. Experimental Section

### 2.1. Chemicals and Materials

Zinc nitrate hexahydrate (Zn(NO_3_)_2_·6H_2_O, 98%, Alfa Aesar, MA, USA), Manganese acetylacetonate (Mn(acac)_2_, AR, Aladdin, Shanghai, China), 2-methylimidazole (2-MeIm, 98%, Thermo Fisher Scientific, MA, USA), Iron (III) 2,4-pentanedionate (Fe(acac)_3_, Alfa Aesar, MA, USA), methanol (Sinopharm Chemical, Shanghai, China), KOH (analytical grade, Sinopharm Chemical, Shanghai, China), Nafion D-521 dispersion (5% *w/w* in water and 1-propanol) (Alfa Aesar, MA, USA), N, N-dimethylformamide (DMF, Sinopharm Chemical, Shanghai, China) were directly used. Distilled water was used in all experiments.

### 2.2. Synthesis of Mn/Fe PSACs

Solution A: 1.314 g (16 mM) 2-MeIm was dissolved in 10 mL methanol and sonicated for 10 min. Solution B: 1.19 g (4 mM) Zn(NO_3_)_2_·6H_2_O, 70.6 mg Fe(acac)_3_ (0.2 mM), and 39 mg Mn(acac)_2_ (0.4 mM) were dissolved in 30 mL methanol and sonicated for 10 min. Solution B was quickly added to solution A and the mixture was stirred for 1 h at room temperature. The mixture was transferred into a 50 mL Teflon-lined stainless-steel autoclave and sealed at 120 °C for 5 h. The mixture was centrifuged and washed with DMF until the supernatant was colorless and then washed with methanol three times and dried at 60 °C under vacuum for 12 h. Then, Mn/Fe SACs were obtained after pyrolysis at 1000 °C under an N_2_ atmosphere for 2 h. Finally, Mn/Fe PSACs were modified with SH-PEG by stirring at room temperature.

### 2.3. Characterization

The crystal structure was determined by X-ray powder diffraction (XRD) (Bruker) equipped with Cu-Kα radiation (λ = 0.154 nm). Transmission electron microscopy (TEM) was recorded using an FEI Tecnai G2 S-Twin with a field emission gun. The high-angle annular dark-field scanning transmission electron microscopy (HAADF-STEM) images were acquired with a Titan 80–300 scanning/transmission electron microscope. Raman shifts were carried out by using a LabRAM Aramis Raman spectrometer instrument. The UV-vis absorption spectra were obtained from the U-3100 spectrophotometer (Hitachi, Tokyo, Japan). The X-ray photoelectron spectra (XPS) were taken on a VG ESCALAB MK II electron spectrometer. Dynamic light scattering (DLS) and Zeta potential were obtained by using a Malvern instrument Zetasizer Nano system (Malvern Instruments, Worcestershire, UK). The electron spin resonance (ESR) spectrum was measured by using Bruker EMXplus Spectrometer System (BrukerBioSpin, Rheinstetten, Germany). The elements’ contents were measured by an inductively coupled plasma mass spectrometer (ICP-MS, Agilent 7500Cx, Agilent Technologies, CA, USA).

### 2.4. Hemolysis and the Shape of RBCs

The centrifugation of whole blood (5 mL, with anticoagulation) at 3000 rpm for 15 min could acquire RBCs. PBS was used to dilute the RBCs (V_PBS_:V_RBCs_ = 9:1). Afterward, Mn/Fe PSACs were confected into different concentration dispersion (2 mL) by PBS, and then the dispersions were incubated with 0.5 mL of RBCs suspension. 2 mL of deionized water and PBS were mixed with 0.5 mL of RBCs suspension, respectively, served as positive and negative controls. Keeping all mixtures maintain at 37 °C for 3 h. After the mixtures were centrifuged at 3000 rpm for 15 min, the optical density (O.D.) of the supernatant at 541 nm was measured by a microplate reader (BioTek synergy 2). The hemolysis rate was calculated with the following formula:

Hemolysis rate % = (O.D._sample_ − O.D._negative_)/(O.D._positive_ − O.D._negative_) × 100% (1)

where O.D._sample_, O.D._positive_, and O.D._negative_ was the O.D. of the sample, positive control, and negative control, respectively. The observation of RBCs’ shape through light microscopy (BM2100, Nanjing Jiangnan Novel Optics Co., Ltd., Nanjing, China).

### 2.5. Decomposition of H_2_O_2_

For verifying the catalytic durability of 200 μg/mL toward H_2_O_2_, H_2_O_2_ solution was added repeatedly to the 200 μg/mL solution followed by measuring the catalytic efficiency under a pH value of 6.5. An optical oxygen sensor (NeoFox, Ocean Optics, FL, USA) was used to quantify the amount of evolving oxygen in the reaction system (150 µM of H_2_O_2_ and 200 μg/mL of Mn/Fe PSACs).

### 2.6. GSH-Depleted Property of Mn/Fe PSACs

The consumption of GSH was detected with a DTNB probe by UV-vis-NIR spectroscopy. The as-obtained Cu SASs/NPC with different concentrations (200 μg/mL) were mixed with GSH (1.0 mM) at room temperature. At different time points, 100 μL of the mixture was added into 900 μL phosphate buffer saline (PBS, pH 7.4), and then DTNB (0.1 mM) was added to the mixed solution. Three minutes later, the absorbance spectrum of this mixed solution was recorded by a UV-vis-NIR spectrophotometer.

### 2.7. POD-Mimic Activity

POD-mimic activity assays of Mn/Fe PSACs were performed using Tetramethylbenzidine (TMB) as substrates in the presence of H_2_O_2_. In brief, Mn/Fe PSACs (200 μg/mL), H_2_O_2_ (1 mM), and TMB (100 μg/mL) were added to 2 mL of PBS solution (pH 6.5) at 42 °C. The absorbance of the color reactions was recorded after a certain reaction time using a UV-vis spectrophotometer.

### 2.8. Oxidase-like Activity of Mn/Fe PSACs and Kinetic Assay

The oxidase-like activity of the Mn/Fe PSACs was tested by oxidation of TMB in the HAc-NaAc buffer solution (0.1 M, pH = 4.5). In the general procedure, 25 µL (200 µg/mL) Mn/Fe PSACs solution was added into 2.0 mL buffer solution, followed by 20 µL TMB (in EtOH, 5 mg/mL). UV–Vis absorption spectra were used to measure the absorbance at 370 and 652 nm. The kinetic assays of nanozyme with TMB as the substrate was performed by adding different amounts (0, 100, 200, 400, and 800 µmol/L) of TMB solution. The Michaelis–Menten constant was calculated according to the Michaelis–Menten saturation curve.

### 2.9. Cell Culture

HepG2 cells were seeded in RPMI 1640 medium supplemented with 10% FBS, penicillin (100 units/mL), and streptomycin (100 μg/mL) in 5% CO_2_ at 37 °C.

### 2.10. Cell Uptake Assay

Mn/Fe PSACs were labeled with Cy5 and centrifuged to eliminate excess free Cy5. HepG2 cells (5 × 10^5^ cells, 24-well plate) were cultured in RPMI 1640 medium at 37 °C. The HepG2 cells were treated with the free Mn/Fe PSACs (20 μg/mL). The HepG2 cells were then incubated for 4 h. At last, PBS was used to wash the cells and a fluorescent microscope was used to observe the cell morphology.

### 2.11. Intracellular Generation of ROS

The HepG2 cells (1 × 10^5^ cells/well) were cultured in a 24-well plate for 1 day. The culture medium was discarded and the fresh culture medium containing Mn/Fe PSACs (200 μg/mL) and H_2_O_2_ (100 μM) was added 4 h later, adding 2,7-dichlorofluorescein diacetate (DCFH-DA) in the medium was incubated for a further 0.5 h. At last, PBS was used to wash the cells and a fluorescent microscope was used to observe the cell morphology.

### 2.12. In Vitro Living/Dead Staining

HepG2 cells were seeded on glass-bottom dishes and cultured for 24 h and transferred with fresh acidified medium (pH = 6.0) containing different samples and H_2_O_2_ (100 μM) and sequentially incubated for another 4 h. After 24 h, cells were washed with PBS for three times and stained with calcein-AM (2 µL) and PI (2 µL) for 15 min. Cells were washed with PBS three times again and detected by using a fluorescent microscope.

### 2.13. In Vitro Cell Cytotoxicity Assay

The cytotoxicity of Mn/Fe PSACs was evaluated in HepG2 cells following the CCK-8 assay standard protocol. Briefly, HepG2 cells were seeded in 96-well plates (5 × 10^3^ cells per well) and cultured in complete RPMI 1640 media for 12 h at 37 °C. Then, media were replaced with fresh media containing Mn/Fe PSACs (0 to 250 μg/mL) and H_2_O_2_ (100 μM) and incubated for 24 h. Afterward, the media was replaced with 100 μL of fresh complete medium containing 10% CCK-8. The absorbance at 450 nm of each well was recorded using a microplate reader (Multiskan MK3, Thermo Scientific, Waltham, MA, USA).

### 2.14. Animal Model

Female nude mice (5–7 weeks old) were obtained from Jiangsu KeyGEN BioTECH Corp., Ltd (Nanjing, China). All performance of in vivo experiments was in line with the institutional animal use and care regulations approved by Jiangnan University (ethics committee approval code, IACUC-001-20). Mice were subcutaneously injected with HepG2 cells.

### 2.15. In Vivo Magnetic Resonance Imaging (MRI)

The intravenous injection of Mn/Fe PSACs (100 μL, 10 mg/kg) in tumor-bearing mice was used to perform in vivo MRI. The MRI photos were obtained through Bruker Icon 3.0 T scanning mice after 24 h.

### 2.16. In Vivo Ultrasound (US) Imaging

The Esaote MyLab Twice device was employed to perform in vivo US imaging. The SAFe-NMCNs nanozyme (100 μL, 10 mg/kg) was intravenously injected in tumor-bearing mice and then captured the image of tumors after 24 h.

### 2.17. In Vivo Antitumor Effects

Tumor-bearing mice were randomly divided into 2 groups with 5 mice per group. Subsequently, mice in every group were intravenously administrated the following treatments (10 mg/kg): (I) PBS, and (II) Mn/Fe PSACs. Body weight and tumor size of mice with different administrations were recorded every two days. Tumor volume (V) was calculated as the following formula: V = width^2^ × length/2. After 14 days, all the mice were sacrificed and tumors and main organs (liver, spleen, kidney, heart, and lung) were collected from every group. Then tumors and major organs were immersed in a 4% paraformaldehyde solution for ready-to-use. Tissues were embedded in paraffin and sectioned into slices, then undertaken to hematoxylin and eosin (H&E) staining, Ki-67 immunohistochemistry staining, and TdT-mediated dUTP-biotin nick end labeling (TUNEL) staining assay.

### 2.18. Statistical Analysis

All the test data in this work were calculated mean value and standard deviation (mean ± S.D.). The statistical analysis of experimental groups was compared by the Student’s *t*-test. *p* < 0.05 (*), *p* < 0.01 (**), and *p* < 0.001 (***) were regarded as statistically significant.

## 3. Result and Discussion

**Figure 1:**
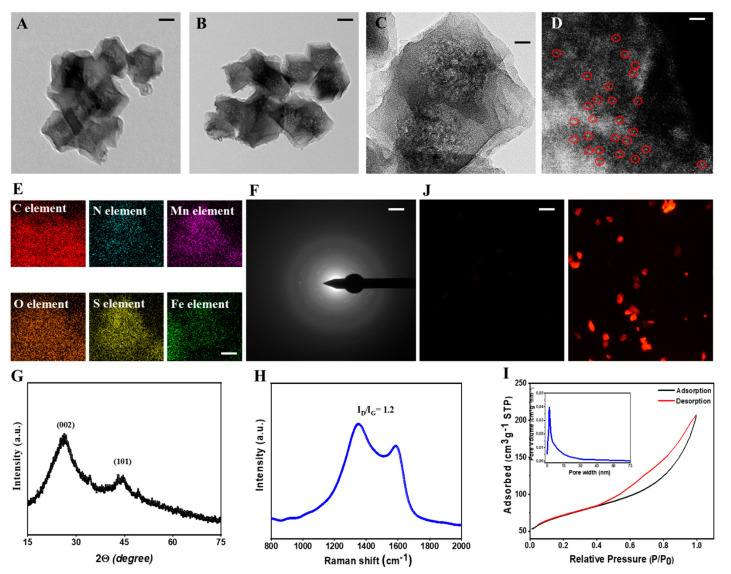
TEM images of (**A**) Mn/Fe ZIF-8s and (**B**) Mn/Fe SACs. Scale bar, 50 nm. (**C**) HRTEM image of single Mn/Fe SACs. Scale bar, 5 nm. (**D**) Representative magnified AC HAADF-STEM image of Mn/Fe SACs. The dispersive single atoms as bright dots (red circles). Scale bar, 2 nm. (**E**) The elemental mapping of Mn/Fe SACs. Scale bar, 15 nm. (**F**) SAED pattern of Mn/Fe SACs. Scale bar, 1 nm. (**G**) XRD pattern of Mn/Fe SACs. (**H**) Raman spectrum of Mn/Fe SACs. (**I**) N_2_ sorption isotherms of Mn/Fe SACs. The inset exhibits the pore size distribution of Mn/Fe SACs. (**J**) Microscopy images of Mn/Fe SACs and Cy5.5-PEG@Mn/Fe SACs. Scale bar, 500 nm.

Mn/Fe SACs were synthesized using a “top-down” approach, which achieved stripping metal nanoparticles (NPs) into a single atom [38,39]. As usual, Fe and Mn ions could be encapsulated into ZIF-8s to form Mn/Fe ZIF-8s. As shown by transmission electron microscopy (TEM), Mn/Fe ZIF-8s had a uniform rhombohedral dodecahedral geometry with a particle size of 90 nm (Figure 1A). Then, Mn/Fe ZIF-8s was performed, followed by pyrolysis at 800 °C for 3 h under an N_2_ atmosphere. The morphology of Mn/Fe SACs was identified by TEM without large crystalline NPs (Figure 1B). Furthermore, the high-resolution TEM (HRTEM) images showed that a single Mn/Fe SAC structure had obvious graphite stripes (Figure S1) and rich pores (Figure 1C). The aberration-corrected high-angle annular dark-field scanning transmission electron microscopy (AC HAADF-STEM) image with atomic resolution predicted the successful anchoring of isolated Mn/Fe atoms in red circles on the carbon matrix (Figure 1D). Uniform distributions of Mn, Fe, and N atoms were verified by energy-dispersive X-ray spectroscopy (EDS) enabled elemental mapping (Figure 1E). Meanwhile, the typical TEM-EDX point-detection of the composition also confirmed the simultaneous existence of Fe and Mn elements (Figure S2). The characteristic diffuse halo in the selected area electron diffraction spectrum (SAED) further demonstrated the fully amorphous nature, with no visualized crystalline Fe and Mn NPs (Figure 1F).

XRD pattern characterized two broad peaks at (002) and (101) for graphitic carbon in Mn/Fe SACs, in the range of 20−30° and 40−50°, respectively (Figure 1G). Raman spectrum of Mn/Fe SACs exhibited two typical peaks of D and G bands in graphitized carbon around 1390 cm^−1^ and 1530 cm^−1^, respectively (Figure 1H). The mesoporous structure of Mn/Fe SACs was predicted by the typical type-IV curves in the N_2_ adsorption−desorption isotherms (Figure 1I). The surface areas of Mn/Fe SACs were calculated to be 227.8 m^2^/g with uniform pore sizes of 3.6 nm by Brunauer−Emmett−Teller (BET) (inset of Figure 1I). The high BET value of Mn/Fe SACs was attributed to the introduction of Fe and Mn species, which increased the microporosity. The binding states of contained elements (Mn, Fe, and N) in Mn/Fe SACs were detected through XPS (Figure S3A). As shown in Figure S3B, the high-resolution Fe 2p spectrum had two peaks at 710.6 and 723.9 eV, which were from the Fe 2p_3/2_ and Fe 2p_1/2_ orbits, illustrating the active sites of Fe in Mn/Fe SACs. Meanwhile, the two peaks in the high-resolution Mn 2p spectrum at 710.4 and 724.1 eV, which were from the Mn 2p_3/2_ and Mn 2p_1/2_ orbits, illustrate the Mn active sites in Mn/Fe SACs (Figure S4A). As shown in the schematic diagram in Figure S4B, the high-resolution N 1s spectrum of Mn/Fe SACs could be deconvoluted into four peaks at approximately 398.2, 399.4, 400.3, and 403.9 eV, corresponding to pyridinic–N, Fe–N*x*, Mn–N*x*, graphitic-N, and oxidized–N. The Mn and Fe concentration in Mn/Fe SACs was quantified to be 0.28 wt % and 0.72 wt % via ICP-MS (Table S1).

**Figure 2:**
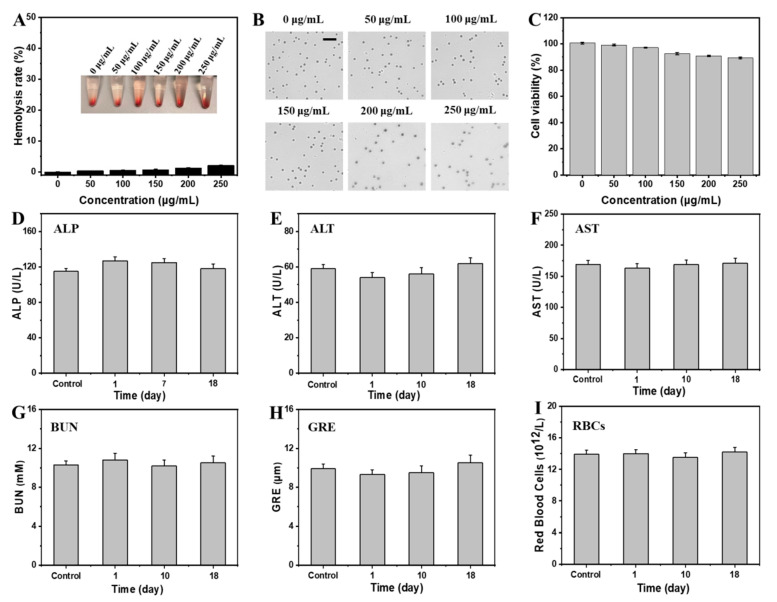
(**A**) Hemolysis rate of RBCs under different concentrations of Mn/Fe PSACs. Inset is the RBCs supernatant images. (**B**) Morphology of RBCs after different treatments. Scale bar, 50 μm. (**C**) Cell viability of cells incubated with different concentrations of Mn/Fe PSACs. (n = 3). (**D**–**I**) Blood routine parameters of mice after Mn/Fe PSACs treatment for 18 days. (n = 3).

To endow Mn/Fe SACs with better biocompatibility, SH-PEG was modified on the surface of Mn/Fe PSACs by electrostatic adsorption. SH-PEG was labeled with the red fluorescent agent cyanine 5.5 (Cy5.5) to predict the successful modification on the surface of Mn/Fe SACs by a confocal laser scanning microscope (CLSM). Unlike the general darkness of Mn/Fe SACs, Cy5.5-PEG@Mn/Fe SACs exhibited distinct red fluorescence (Figure 1J). The obtained PEGylated Mn/Fe SACs (Mn/Fe PSACs) could be well dispersed in various physiological conditions (Figure S5). The measured values of average hydrodynamic size and polymer dispersity index (PDI) showed no changes, and the stability characteristics were further verified by ICP-MS to detect leakage of Fe and Mn leaching during incubation (Figure S6). As a qualified candidate for biomedical applications, the biocompatibility of NPs needs to be assessed beforehand. Herein, the hemolysis rate was analyzed to assess the blood compatibility of Mn/Fe PSACs with rat red blood cells (RBCs). The highest hemolysis rate after Mn/Fe PSACs (250 μg/mL) incubation was less than 5% (Figure 2A), which excluded the hemolytic probability of Mn/Fe PSACs as intravenously administrating agents. Representative microscopy images also confirmed that the morphology of RBCs did not change between different groups (Figure 2B).

Based on the above data, we concluded that Mn/Fe PSACs are suitable for drug delivery systems and other in vivo treatments. HepG2 cell viability was tested by the CCK-8 method to determine the toxicity of Mn/Fe PSACs, which showed no toxicity with relatively high levels of cell viability in various treatments (Figure 2C). In addition, in vivo hematological analysis was performed to assess the long-term toxicology of Mn/Fe PSACs by collecting blood samples at predicted time points (days 10th and 18th) after intravenous injection into nude mice (Figure 2D–I). Blood samples on the day 1st were collected before injection. The general blood routine parameters of the Mn/Fe PSACs-injected mouse samples were not significantly different, indicating that the hepatotoxic effect of Mn/Fe PSAC injection was negligible.

**Figure 3:**
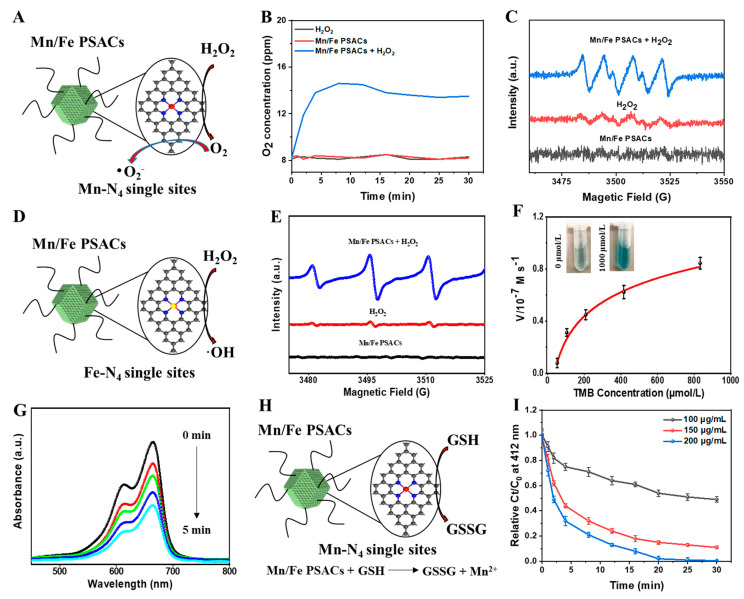
(**A**) Schematic illustration of the •O_2_^−^ catalytic activity of Mn/Fe PSACs. (**B**) The O_2_ generation from the Mn/Fe PSACs induced the decomposition of H_2_O_2_. (**C**) ESR spectra of •O_2_^−^ trapped by BMPO upon addition of DMSO. (**D**) Schematic illustration of the •OH catalytic activity of Mn/Fe PSACs. (**E**) ESR spectra of •OH trapped by DMPO in the presence of SOD. (**F**) TMB analysis of the generated •OH from Mn/Fe PSAC solutions. (n = 3). (**G**) UV-vis absorption spectra of various reactions with the MB. Lines from top to bottom represent 0 min, 1 min, 2 min, 3 min, and 5 min. (**H**) Schematic illustration of the GSH catalytic activity of Mn/Fe PSACs. (**I**) GSH consumption after Mn/Fe PSACs treatment.

Catalase (CAT) is an enzyme that can catalyze the decomposition of H_2_O_2_ to generate O_2_ (Figure 3A) [40,41]. In Figure 3B, with the increasing O_2_ concentration in solution, Mn/Fe PSACs were demonstrated to have similar CAT activity to native catalase in converting H_2_O_2_ into O_2_. Based on the oxidase (OXD)-like activity, Mn/Fe PSACs could facilitate electron transfer to substrate O_2_ and generate superoxide radicals (•O_2_^−^). ESR spectroscopy was used to confirm the ROS species by using a •O_2_^−^ trapping agent 5-tert-butoxycarbonyl-5-methyl-1-pyrroline N-oxide (BMPO). The characteristic peaks 1:1:1:1 of BMPO-OOH verified that the generation of •O_2_^−^ was catalyzed by the common CAT- and OXD-like enzymatic activities of Mn/Fe PSACs (Figure 3C).

As exhibited in Figure 3D, the POD activity of Mn/Fe PSACs was demonstrated by detecting the toxic •OH produced by the decomposition of H_2_O_2_ through a Fenton-like reaction. The generation of •OH was characterized by a characteristic quadruple signal (1:2:2:1) of DMPO-OH using a spin trapper 5,5-Dimethyl-1-pyrroline N-oxide (DMPO) (Figure 3E). This result directly demonstrated the POD-like activity of Mn/Fe PSACs from the enzymatic assays described above. The generation of •OH catalyzed by Mn/Fe PSACs was measured by TMB assay. The appearance of blue TMB oxide during the incubation of Mn/Fe PSACs with H_2_O_2_ increases with increasing TMB concentration (Figure 3F). The K_M_ value of the nanozyme was 0.079 mM for the TMB. The corresponding V_max_ value is 6.17 × 10^−8^ M s^−1^. In addition, the POD-like activity of Mn/Fe PSACs was enhanced in a mildly acidic pH environment, which demonstrated that Mn/Fe PSACs could initiate more pronounced POD activity in TME (Figure S7). Moreover, the POD-like catalytic properties of Mn/Fe PSACs were further tested with methylene blue (MB) probes (Figure 3G) and o-phenylenediamine (OPDA) (Figure S8). Similar phenomena were found using MB probes, fully demonstrating the excellent POD-like catalysis of Mn/Fe PSACs.

As previously mentioned, POD-like nanozymes generated ROS could be depleted by GSH presented in excess in the TME [3,18,20]. Mn-based catalysts have excellent GSH depletion ability due to their multivalent nature, which can effectively rescue the loss of ROS products [42,43]. Therefore, we proposed the mechanism of GSH depletion by Mn/Fe PSACs in Figure 3H. The GSH-depleting capability of Mn/Fe PSACs was evaluated using the 5, 5′-dithio-bis (2-nitrobenzoic acid) (DTNB) probe. It could be found that the characteristic peak of DTNB at 412 nm was significantly reduced under the condition of Mn/Fe PSACs (Figure 3I). As the concentration of Mn/Fe PSACs increased, the depletion of GSH became faster in a concentration-dependent manner. The result showed that Mn/Fe PSACs acted as a GSH peroxidase (GSH-Px)-like mimetic enzymes with satisfactory GSH-consuming ability.

**Figure 4:**
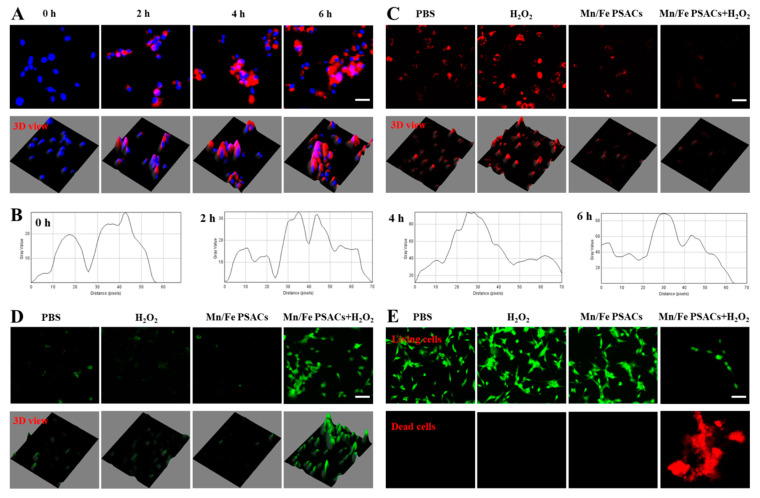
(**A**) Cellular uptake of Mn/Fe PSACs in HepG2 cells at different times. Scare bar, 25 μm. (**B**) The quantitative fluorescence intensity by Image J. (**C**) The intracellular hypoxia imaging using a [Ru(dpp)_3_]_2_Cl_2_ probe. Scare bar, 50 μm. (**D**) Detection of ROS generation in HepG2 cells after different treatments. Scare bar, 50 μm. (**E**) HepG2 cells were co-stained with calcein-AM (green for live cells) and PI (red for dead cells). Scare bar, 50 μm.

Next, the cellular uptake behavior of Mn/Fe PSACs was monitored after labeling NPs with Cy5.5. CLSM showed that Mn/Fe PSACs could be internalized after 2 h of incubation and increased within 6 h (Figure 4A). The intracellular fluorescence intensities in different time points were quantified by Image J. As shown in Figure 4B, the intracellular fluorescence intensity of Cy5.5-Mn/Fe PSACs was proportional to the incubation time within 6 h. Moreover, the common overlap between the red signal from Cy5.5-Mn/Fe PSACs and the green signal from the lysosomes after 2 h of incubation suggested efficient accumulation of Cy5.5-Mn/Fe PSACs in lysosomes (Figure S9). The O_2_ generation ability in cancer cells was also detected by using [Ru(dpp)_3_]Cl_2_, an intracellular O_2_ level indicator with red fluorescence. The fluorescence in cells treated with Mn/Fe PSACs plus H_2_O_2_ was much weaker than that of the PBS control, H_2_O_2_, and Mn/Fe PSACs groups due to the increased intracellular O_2_ level based on Mn/Fe PSACs-induced single atom-based catalysis (Figure 4C). These data collectively confirmed that Mn/Fe PSACs could effectively regulate the in situ H_2_O_2_ decomposition and O_2_ production. Simultaneously, the dual enzyme-mimetic catalytic activity of Mn/Fe PSACs was evaluated in HepG2 cells by measuring the ROS level using a DCFH-DA fluorescent probe. In the presence of ROS, DCFH-DA would be oxidated into 2,7-dichlorofluorescein (DCF) with green fluorescence. As shown inFigure 4D, cells incubated with Mn/Fe PSACs plus H_2_O_2_ exhibited the strongest green fluorescence due to the admirable POD-mimetic catalytic performance of Mn/Fe PSACs, which could catalyze the decomposition of H_2_O_2_ into ROS, while the green signals were negligible in PBS, H_2_O_2_, and Mn/Fe PSACs groups.

**Figure 5:**
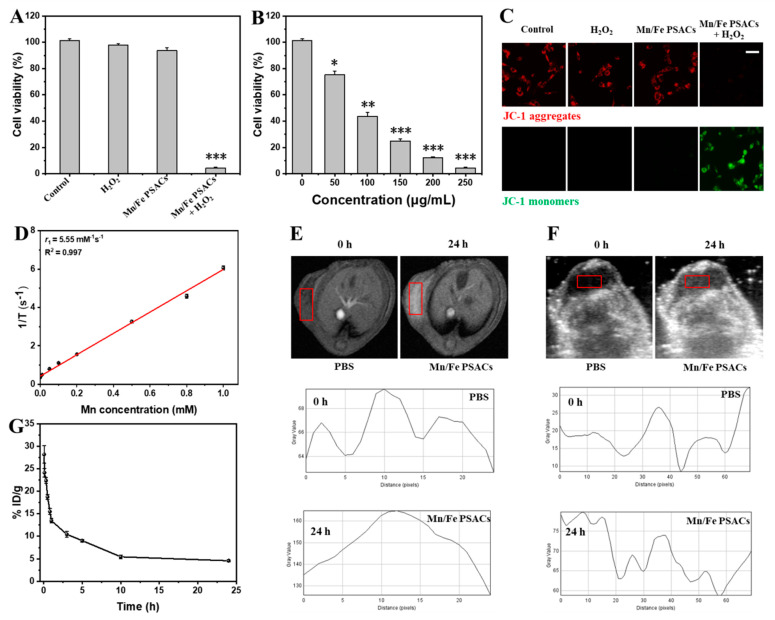
(**A**) Cell viability of cells after different treatments. (*** *p* < 0.001) (**B**) Cell viability of cells with different concentrations of Mn/Fe PSACs. (* *p* < 0.05, ** *p* < 0.01, and *** *p* < 0.001) (**C**) JC-1 analysis of cells after different treatments. Scale bar, 50 μm. (**D**) Transverse relativity (r1) value of different concentrations of Mn icon. (**E**) MRI and (**F**) US imaging of tumor-bearing mice after Mn/Fe PSACs treatment. The signal intensity was quantified by Image J. (**G**) Blood circulation profile of Mn/Fe PSACs in mice after intravenous injection.

The apoptosis of HepG2 cells treated with different treatments were visualized by co-staining with calcein-AM (living cells, green fluoresce) and PI (dead cells, red fluoresce). As shown in Figure 4E, a large number of dead cells appeared in Mn/Fe PSACs to quantify cell viability after different treatments. As shown in Figure 5A, co-cultivation of tumor cells with Mn/Fe PSACs plus H_2_O_2_ greatly reduced cell viability (97.5%) compared to PBS, H_2_O_2_, and single Mn/Fe PSACs treated groups, which was consistent with living/dead staining experiments. To explore the concentration-dependent manner effect of nanozyme-induced cancer cell death, we investigated cell viability by varying the concentration (0, 50, 100, 150, 200, and 250 µg/mL) of Mn/Fe PSACs. In Figure 5B, cell viability significantly decreased with increasing Mn/Fe PSACs concentration, revealing that Mn/Fe PSACs could effectively mediate cancer cell death by inducing the CDT effect. 5,5′,6,6′-tetrachloro-1,1′,3,3′-tetraethyl-imidacarbocyanine iodide (JC-1) was widely used as a fluorescent probe to detect changes in mitochondrial membrane potential, and it could exhibit red fluorescence (from JC-1 aggregates) on intact mitochondrial membranes, and green fluorescence (from JC-1 monomers) on damaged mitochondrial. As shown in Figure 5C, compared with PBS, H_2_O_2_, and Mn/Fe PSACs groups, HepG2 cells co-cultured with the Mn/Fe PSACs plus H_2_O_2_ showed stronger green fluorescence from JC-1 monomers and negligible red fluorescence from JC-1 aggregates. This result confirmed that Mn/Fe PSACs could induce cancer cell apoptosis with the existence of H_2_O_2_ by generating toxic ROS to damage the mitochondrial membrane.

To illustrate the stimuli-responsive MRI properties, the T1-weighted MRI images and longitudinal relaxivity (r1) of the Mn/Fe PSAC solutions with different Mn concentrations were evaluated using 3.0 T clinical MRI equipment. T1-weighted magnetic resonance images of Mn/Fe PSACs showed concentration-dependent signal enhancement (Figure 5D). The corresponding longitudinal relaxivity (r1) value was quantitatively calculated to be 5.55 mM^−1^ s^−1^, which was higher than that of clinically Gd-based contrast agents (Magnevist, r1 = 4.56 mM^−1^ s^−1^). Thereafter, we utilize those samples to perform a T1-weighted MRI in a tumor-bearing mouse model. Before and after (24 h distribution) Mn/Fe PSAC (15 mg/kg) i.v. injection. The MRI signal intensity of Mn/Fe PSACs in the tumor area was significantly enhanced. Quantitative MRI signals within Mn/Fe PSACs-treated tumor sites further corroborate these findings (Figure 5E). To confirm that the CAT-mimetic catalytic performance of Mn/Fe PSACs could achieve US imaging in vivo, mice were injected with Mn/Fe PSACs intravenously and then monitored by US imaging at different time points. Compared with pre-injection, US imaging contrast of the tumor area tended to be enhanced after Mn/Fe PSACs injection. Quantitative US signals within the tumor sites treated by Mn/Fe PSACs further corroborate these findings (Figure 5F). The increase in US signals was mainly because Mn/Fe PSACs could decompose intratumoral H_2_O_2_ and generate O_2_ bubbles through CAT-mimicking catalytic activity, thereby achieving impedance mismatch and generating sufficient echogenic reflectivity. These imaging abilities of the Mn/Fe PSACs would be very useful for guiding in vivo therapy. Pharmacokinetics studies showed that Mn/Fe PSACs had a long elimination half-life (Figure 5G), indicating that Mn/Fe PSACs exhibited a satisfactory blood retention potential over an examination period of 24 h.

**Figure 6:**
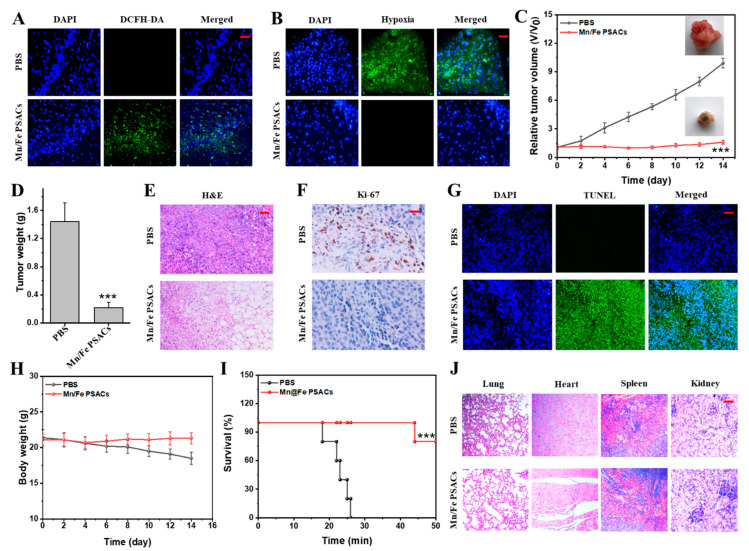
Detection of (**A**) ROS generation and (**B**) hypoxia in tumor tissues after different treatments. Scale bar, 50 μm. (**C**) Relative tumor volumes and (**D**) weight of tumors of mice from different groups. (*** *p* < 0.001) (**E**) H&E, (**F**) Ki-67 staining analyses, and (**G**) TUNEL fluorescent images of tumor tissues after different treatments. Scale bar, 50 μm. (**H**) The body weight of mice after different treatments. (**I**) The survival rate of mice after different treatments. (*** *p* < 0.001) (**J**) H&E staining images of main organs after different treatments. Scale bar, 50 μm.

To test the activation of the nanoreactors specific to tumor sites, we first monitored ROS levels in living mice using CLSM. After 24 h post-injection of Mn/Fe PSACs and control groups, the ROS probe (DCFH-DA) was intravenously injected, and the green fluorescence intensity in the tumor was monitored using dorsal window chamber models due to the ROS-responsive green fluorescence conduction characteristics of the probe. As shown inFigure 6A, strong green fluorescence in the Mn/Fe PSACs group indicated a sharp increase in ROS levels in tumor tissues. Moreover, immunofluorescence staining was performed to confirm the capability of Mn/Fe PSACs for in situ amelioration of hypoxia status within the tumor. To this end, pimonidazole hydrochloride was first employed as a hypoxia probe to detect the hypoxia state of tumor tissues. Tumor hypoxia and blood vessels were then stained with an anti-pimonidazole antibody (green signals). The results showed that mice treated with Mn/Fe PSACs could significantly reduce hypoxia signals in tumor tissues, while the control group exhibited bright green hypoxia signals (Figure 6B). It could be concluded that the tumor hypoxia was alleviated due to the catalysis of H_2_O_2_ into O_2_ in the presence of Mn/Fe PSACs.

Subsequently, we explored the antitumor effects of the combined enzymatic properties of Mn/Fe PSACs. The treatment procedure was as follows: Mn/Fe PSACs were injected into tumor-bearing BALB/c nude mice at the dose of 15 mg/kg. Interestingly, Mn/Fe PSACs significantly inhibited tumor growth during the treatment period for 14 days (Figure 6C). At the end of treatment, mice from all groups were euthanized and tumor tissues were harvested. The results showed that the average weight of anatomically obtained tumors in the Mn/Fe PSACs group was only 0.21 g (Figure 6D). H&E staining analysis of tumor tissues revealed severe necrosis in Mn/Fe PSACs treated mice, where nuclear condensation and cell shrinkage occurred (Figure 6E). Furthermore, Ki-67 and TUNEL staining were performed on tumor sections to study the proliferation and apoptosis levels of tumor cells, respectively. Mn/Fe PSACs treatment group had the least brown area in the tumor section, indicating that it had a good inhibitory effect on tumor cell proliferation (Figure 6F). Similarly, the results of the TUNEL analysis also showed that there were more apoptotic cells in the Mn/Fe PSACs-treated group compared with the control group (Figure 6G). Systemic toxicity of Mn/Fe PSACs was evaluated by monitoring changes in body weight and survival rate during treatment. There was no significant change in body weight between different treatment groups, indicating that Mn/Fe PSACs treatment did not affect the growth of mice (Figure 6H). All mice in the control group died within 27 days. In contrast, mice in the Mn/Fe PSACs group survived for more than 44 days (Figure 6I), manifesting significantly elevated survival rates. Moreover, an H&E staining assay was performed on major organs (heart, lung, spleen, and kidney) after drug administration. No visible damage was observed in the main organs, indicating the good biocompatibility of Mn/Fe PSACs (Figure 6J). These results confirmed that the Mn/Fe PSACs could exert a superior inhibitory effect on tumor cells through CDT with minimal side effects.

## 4. Conclusions

In conclusion, dual POD-like and GSH-like nanozyme-Mn/Fe PSACs were synthesized for catalytic antitumor therapy. The doping of Fe and Mn single atoms significantly enhanced the POD-like catalytic activity and GSH-depleting function. This multifunctional single-atom enzyme catalyzes a cascade of reactions that harnesses the high levels of H_2_O_2_ in the TME to efficiently generate ROS to kill cancer cells. The CAT-like function of Mn/Fe PSACs catalyzed the decomposition of H_2_O_2_ and generated a large amount of O_2_ in cancer cells. Subsequently, the OXD-like function mediated electron transfer to O_2_, which generates large amounts of cytotoxic •O_2_^−^, thereby inducing apoptosis. The POD-like activity of Mn/Fe PSACs catalyzed •OH generation via H_2_O_2_ decomposition. Upon internalization by tumor cells, the catalytic activity of Mn/Fe PSACs was significantly elevated by local weakly acidic conditions. In vitro and in vivo acute toxicity evaluation experiments proved that Mn/Fe PSACs had good biosafety and could be used safely for intravenous administration. Moreover, O_2_ and ROS had been demonstrated to be abundantly produced in tumor cells. In vivo and in vitro cancer treatment results showed that Mn/Fe PSACs could effectively kill tumor cells, thus possessing excellent CDT therapeutic ability. Taken together, Mn/Fe PSACs can serve as a tumor-specific activatable nanomedicine against liver cancer with significant potential for clinical translation.

## Data Availability

Data are available on request.

## Supplementary Materials

The following supporting information can be downloaded at: https://www.mdpi.com/article/10.3390/pharmaceutics15041217/s1, Figure S1. HR-TEM image of Mn/Fe SACs. Scale bar, 1 nm. Figure S2. EDX spectrum of Mn/Fe SACs. Figure S3. (A) XPS survey scan and (B) Fe 2p spectra of Mn/Fe SACs. Figure S4. (A) Mn 2p and (B) N 1s spectra of Mn/Fe SACs. Figure S5. The distribution of Mn/Fe PSACs in a different medium for 7 days. Figure S6. The (A) size and (B) PDI changes of Mn/Fe PSACs in a different medium. Figure S7. TMB assay for measuring oxidase-like activity of the Mn/Fe PSACs at the different pH. Figure S8. UV–vis–NIR spectra of OPDA oxidized by Mn/Fe PSACs in the presence of H_2_O_2_ after different times. Figure S9. Colocalization of lysosome with Mn/Fe PSACs after co-incubation for 2 h. Table S1. ICP-MS results for Mn/Fe SACs.
