# Supplementary material for: Stimuli-Responsive Double Single-Atom Catalysts for Parallel Catalytic Therapy
Source: Pharmaceutics. 2023 Apr 11;15(4):1217. doi: 10.3390/pharmaceutics15041217 (PMC10143931; doi:10.3390/pharmaceutics15041217)
Supplement: Supplementary file 1 [file pharmaceutics-15-01217-s001.zip › pharmaceutics-2332861-supplementary.pdf]

Supporting Information

Stimuli-Responsive Double Single-Atom Catalysts for Parallel Catalytic Therapy

Supporting figures

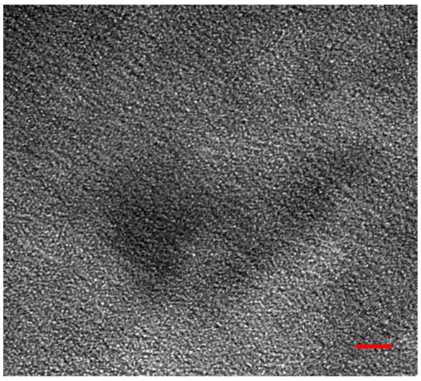

Figure S1. HR-TEM image of Mn/Fe SACs. Scale bar, 1 nm.

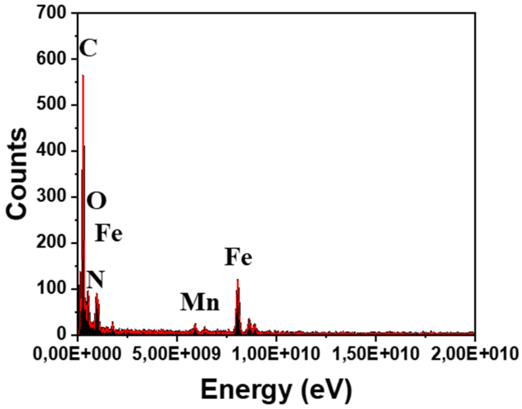

Figure S2. EDX spectrum of Mn/Fe SACs.

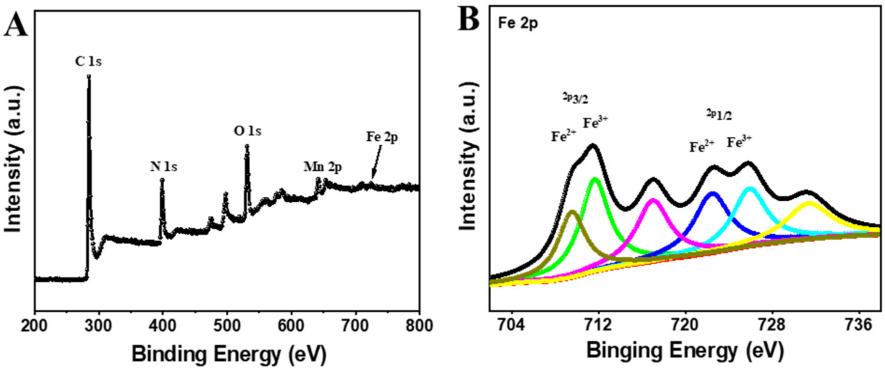

Figure S3. (A) XPS survey scan and (B) Fe 2p spectra of Mn/Fe SACs.

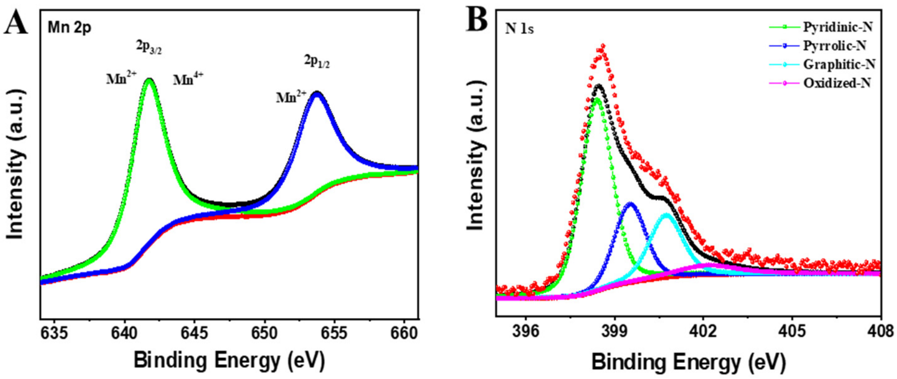

Figure S4. (A) Mn 2p and (B) N 1s spectra of Mn/Fe SACs.

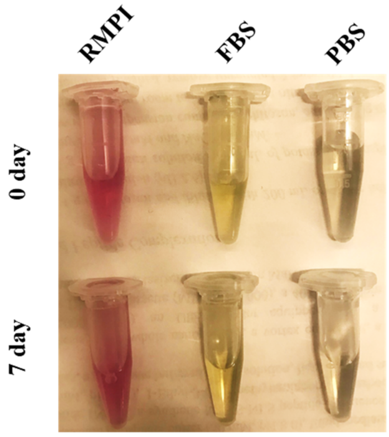

Figure S5. The distribution of Mn/Fe PSACs in a different medium for 7 days.

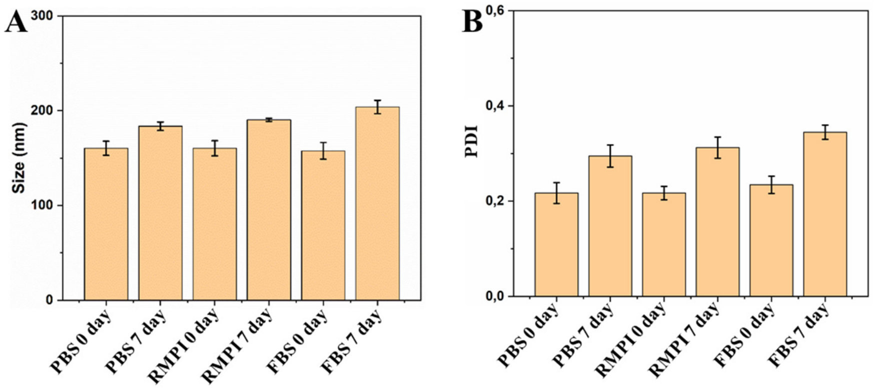

Figure S6. The (A) size and (B) PDI changes of Mn/Fe PSACs in a different medium.

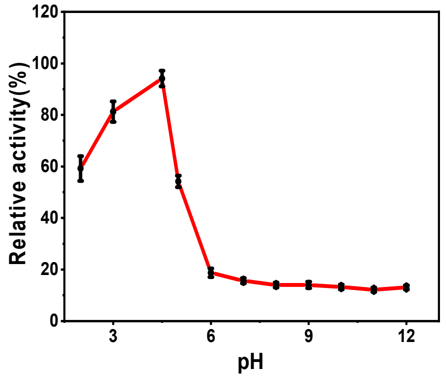

Figure S7. TMB assay for measuring oxidase-like activity of the Mn/Fe PSACs at the different pH.

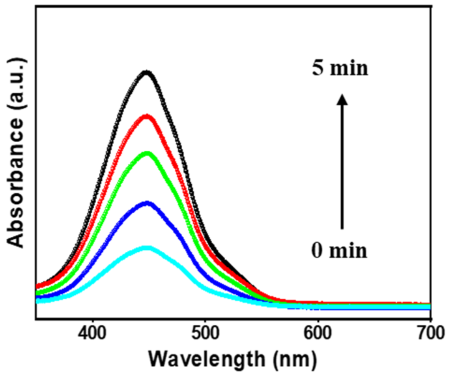

Figure S8. UV–vis–NIR spectra of OPDA oxidized by Mn/Fe PSACs in the presence of H_2O_2 after different times.

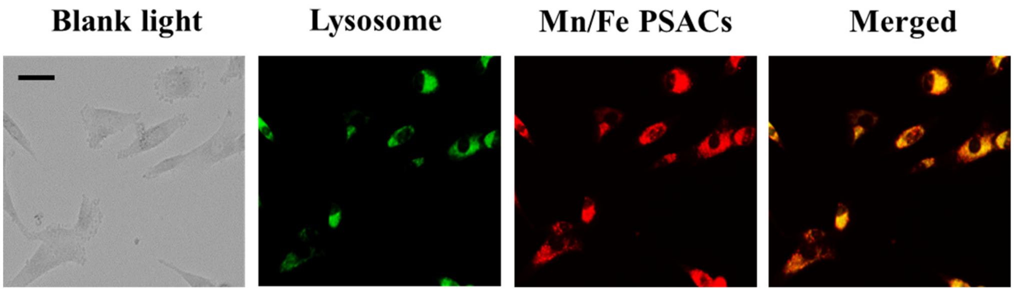

Figure S9. Colocalization of lysosome with Mn/Fe PSACs after co-incubation for 2 h.

Table S1. ICP-MS results for Mn/Fe SACs.

Sample	Element	Content (%)
Mn/Fe PSACs	Mn	0.28
	Fe	0.72
